# Three new species of *Uvariodendron* (Annonaceae) from coastal East Africa in Kenya and Tanzania

**DOI:** 10.3897/phytokeys.174.61630

**Published:** 2021-03-12

**Authors:** Léo-Paul M.J. Dagallier, Frank M. Mbago, W.R. Quentin Luke, Thomas L.P. Couvreur

**Affiliations:** 1 DIADE, Univ Montpellier, IRD, CIRAD, Montpellier, France Univ Montpellier Montpellier France; 2 The Herbarium, Botany Department, Box 35060, University of Dar es Salaam, Dar es Salaam, Tanzania University of Dar es Salaam Dar es Salaam Tanzania; 3 East African Herbarium, National Museums of Kenya, P. O. Box 45166 00100, Nairobi, Kenya National Museums of Kenya Nairobi Kenya; 4 Pontificia Universidad Católica del Ecuador, Facultad de Ciencias Exactas y Naturales Av. 12 de Octubre 1076 y Roca, Quito, Ecuador Pontificia Universidad Católica del Ecuador Quito Ecuador

**Keywords:** Annonaceae, bergamot, Dzombo Hill, endemic, IUCN conservation status, Shimba Hills

## Abstract

East Africa is a hotspot of biodiversity with many endemic plant species. We describe three new species of the genus *Uvariodendron* (Annonaceae) from the coastal forests of Kenya and Tanzania. *Uvariodendron mbagoi* Dagallier & Couvreur, **sp. nov.** is endemic to Tanzania and unique within the genus by its strong bergamot scent and its tomentose fruits having regular tufts of higher hair density. *Uvariodendron dzomboense* Dagallier, W.R.Q. Luke & Couvreur, **sp. nov.** is endemic to Dzombo Hill in Kenya and is rendered distinct by its small leaves and very densely pubescent carpels. *Uvariodendron schmidtii* W.R.Q. Luke, Dagallier & Couvreur, **sp. nov.** is endemic to Shimba Hills in Kenya and differs by its small flowers and fused sepals forming a ring. Following IUCN criteria we assessed *U. mbagoi* and *U. dzomboense* as endangered (EN) while *U. schmidtii* is assessed as Vulnerable (VU). We also propose a new combination: *Polyceratocarpus oligocarpus* (Verdc.) Dagallier, **comb. nov.** The description of these three new species underlines the richness in endemics in East Africa and that new discoveries might arise from further botanical exploration of this region.

## Introduction

East Africa is one of the richest regions in terms of biodiversity across the continent (Myers et al. 2000; Linder 2001). Recently, this region has been described as acting both as a “cradle” (i.e. promoting lineage divergence) and as a “museum” of diversity (i.e. maintaining old lineages), due to its topographical heterogeneity (Dagallier et al. 2020). East Africa harbors an incredible number of endemic species, particularly in the Eastern Arc Mountains and in coastal forests (Burgess et al. 1998, 2007; Küper et al. 2004). Despite the completion of the Flora of East Africa series (Beentje 2015), East Africa still needs further botanical exploration (Sosef et al. 2017). Indeed, from animals (Huber and Warui 2012) to plants (Poulsen and Lock 1997; Friis et al. 2015), new taxa continue to be described.

Annonaceae is a pantropical family of trees, shrubs and lianas. It is the most species rich family within the order Magnoliales, with ca. 2400 recognized species (Chatrou et al. 2012). In East Africa, several new species have been described for the region following the publication of the Flora of Tropical East Africa (Vollesen 1980; Verdcourt and Mwasumbi 1988; Johnson et al. 1999; Deroin and W.R.Q. Luke 2005; Couvreur et al. 2006; Couvreur and W.R.Q. Luke 2010; Marshall et al. 2016; Johnson et al. 2017; Gosline et al. 2019).

The genus *Uvariodendron* contains a total of 14 species restricted to tropical Africa (Fries 1930; Le Thomas 1967, 1969; Verdcourt 1969, 1986). It belongs to the Monodoreae tribe (Chatrou et al. 2012) and was inferred to be the sister genus to the *Uvariopsis* – *Monocyclanthus* clade based on molecular data (Couvreur et al. 2008; Guo et al. 2017). Like many Annonaceae species, *Uvariodendron* species are trees with simple hair indumentum and palgiotropic branches on an orthotropic axis. Their leaves are distichous, simple and entire, with the midrib sunken above, raised below, the secondary veins weakly brochidodromous to brochidodromous and the tertiary veins reticulate. They have hermaphroditic flowers with one whorl of three valvate to imbricate sepals and two whorls of three free and valvate petals. *Uvariodendron* species don’t have a single synapomorphy that can differentiate them from other Annonaceae at first sight, but they can be recognized by the combination of the several characters presented hereafter. The inflorescence is axillary or on the trunk, composed of one to three sessile flowers or with a short pedicel (generally less than 5 cm). The sepals are smaller than, and morphologically different to, the petals. The outer and inner petals are subequal in length, from 10 to 40 mm at anthesis; the outer petals are valvate all along the margin whereas inner petals are valvate only at the apex. The stamens are numerous (more than 200), with linear anthers and truncate connective. The carpels are free, linear, with a coiled stigma. The monocarps are sessile or subsessile and cylindrical.

Here we describe three new species of *Uvariodendron*, from coastal forests in Kenya and Tanzania. We also transfer the species known as *Uvariodendron oligocarpum* Verdc. within the genus *Polyceratocarpus* Engl. & Diels as *Polyceratocarpus oligocarpus* (Verdc.) Dagallier. This brings the number of *Uvariodendron* species up to nine for East-Africa, and 17 for the genus as a whole. A key to East-African *Uvariodendron* species is also presented.

## Material and methods

We examined all the 35 herbarium specimens cited in the results. Among them, we measured 12 specimens for *Uvariodendron mbagoi* (three of which were also examined and measured as living individuals), three herbarium specimens for *Uvariodendron dzomboense*, and four herbarium specimens for *Uvariodendron schmidtii*. Herbarium specimens came from B, DSM, EA, K, MPU, MO, P, and WAG. The three new species are morphologically close to *Uvariodendron kirkii* Verdc., one of the other *Uvariodendron* species occurring in East Africa. In order to ease the discrimination between the species, we present a comparison table of the most discriminant characters between the four species (Table 1). The data for *Uvariodendron kirkii* is taken from Verdcourt (1971) and from more than 50 specimens examined in the above-mentioned herbaria.

**Table 1.** Comparison of the main characters used to discriminate the described species with *Uvariondendron kirkii*. In bold: character unique to the species.

Species	*U. kirkii*	*U. dzomboense*	*U. mbagoi*	*U. schmidtii*
Scent	none reported	none reported	**strong, bergamot**	none reported
Lamina length (mm)	86–210	65–132	76–157	159–188
Leaves margins	flat	slightly revolute	slightly revolute	flat
Pedicel length (mm)	5–28	8–30	**0–0.6**	10– 15
Sepals	free, valvate to imbricate	fused at base	free, imbricate	**fused, forming a ring**
Petals length (mm)	12–39	16–18	unknown on mature flower	**10–12**
Number of carpels	7–20	**50**–**75**	12–16	< 10

For morphological descriptions, we followed the terminology developed by Hickey et al. (1999) and by the Systematics Association Committee for Descriptive Biological Terminology (1962) for leaf and plane shapes, by Payne (1978) for the indumentum, and by Harris and Harris (2001) for the other terms.

The identification key was built with the help of Xper^3^ comparison tools (http://www.xper3.fr/, Vignes Lebbe et al. 2016).

To make a preliminary conservation status assessment for each species, we calculated the extent of occurrence (EOO) and the area of occupancy (AOO) using the ConR package (Dauby et al. 2017). When calculation of EOO and AOO was impossible due to imprecision of coordinates, as for *Uvariodendron dzomboense* and for *Uvariodendron schmidtii*, we calculated the area of the locality in which they occur (respectively the forested part of Dzombo Hill and the Longomwagandi forest) based on Google satellite images with the surface calculation tool in QGIS v. 2.18.17 (QGIS Development Team 2016). We then assigned a preliminary conservation status following IUCN Red List Categories and Criteria Version 3.1 (IUCN 2012).

The distribution map was plotted using *ggmap* (https://CRAN.R-project.org/package=ggmap) package in R (R Core Team 2016). Data on protected areas was taken from Protected Planet (https://www.protectedplanet.net/, accessed June 2018).

## Results

### Uvariodendron mbagoi

Taxon classificationPlantaeMagnolialesAnnonaceae

Dagallier & Couvreur sp. nov.

2976C7E0-5702-539E-AA38-491AF3B2B704

urn:lsid:ipni.org:names:77215717-1

Fig. 1

#### Type.

Tanzania – Tanga • L.-P.M.J. Dagallier 39 (holotype: MPU (MPU1375316), isotypes: DSM, K, MO, MPU (MPU1375317), P, WAG); Handeni District, Kwedijela forest, ca. 8 km from Kwamsisi village; 5°54'50.12"S, 38°36'12.35"E; alt. 156 m; 13 Nov. 2019.

#### Diagnosis.

Differs from other *Uvariodendron* species by its stiff greyish–green leaves with slightly revolute margins, the strong bergamot scent (the citrusy smell of *Citrus bergamia* Risso, between lemon and orange scent) of crushed leaves and bark, its globose flower buds easily falling off and its tomentose fruits having regular tufts of higher hair density. Differs from *Uvariodendron kirkii* by having smaller leaves when looking at the greater leaves (157 mm maximum vs. 210 mm maximum) (Table 1).

#### Description.

Tree or shrub 3–6 m tall, 5–10 cm in diameter at breast height (d.b.h.), slash with strong bergamot smell (the citrusy smell of *Citrus bergamia* Risso); young branches sparsely pubescent to glabrous, old branches glabrous. Leaves distichous, simple, entire, margins slightly revolute, stiff, greyish–green. Petiole 3–6.5 mm long, 1.2–3 mm in diameter, young petiole sparsely pubescent to glabrous, old petiole glabrous. Leaf lamina 76–157 mm long, 31–59 mm wide, length:width ratio 2.2–3.5, narrowly elliptic to elliptic to narrowly obovate, between coriaceous and cartilaginous, apex acute to shortly acuminate, acumen 5–10 mm long, base acute to slightly decurrent (sometimes cuneate), above glabrous, below sparsely pubescent to glabrous when young, glabrous when old; mid rib sunken above, raised below, above glabrous when young and old, below sparsely pubescent to glabrous when young, glabrous when old; secondary veins 10–14 pairs, weakly brochidodromous, indistinct to slightly impressed above, slightly raised to raised below, inter–secondary veins absent; tertiary veins reticulate. Inflorescence borne on trunk or old branches, of 1–2 (3) flowers. Flower sessile or subsessile, pedicel 0–0.6 mm long, 2 mm in diameter. Flowers actinomorphic, hermaphroditic, buds globose 5–9 mm in diameter, velutinous, falling off very easily. Only flower buds and old fallen flowers seen. Bracts 2–5, at base of the pedicel, upper bract 5–8 mm long, 10–15 mm wide, appressed, enclosing bud, pubescent outside, glabrous inside. Sepals 3, ca. 7–8 mm long, ca. 7–12 mm wide (measures taken from bud), imbricate, enclosing the petals in bud, velutinous outside, glabrous inside. Outer petals 3, ca. 4 mm long, ca. 4 mm wide (measures taken from bud). Inner petals 3, ca. 5 mm long, ca. 5 mm wide (measures taken from bud), shortly velutinous outside, glabrous inside. Stamens more than 400, mature length unknown, anthers linear, connective truncate. Carpels 12–16, ca. 1.5 mm long, ca. 1 mm wide (measures taken from old flower), velutinous, stigma coiled. Fruiting pedicel 0–6 mm long, ca. 4 mm in diameter, pubescent. Monocarps 1–7, 20–50 mm long, 10–12 mm wide, length:width ratio 2–4.5, cylindrical, generally curved, showing constrictions and longitudinally ridged, green–grey, tomentose with regular tufts of higher hair density, shortly stipitate, stipe 0–1.5 mm long, 5 mm wide, tomentose. Seeds 4–17 per monocarp, uniseriate to biseriate, 8–8.5 mm long, 5.5–6 mm wide, glabrous.

**Figure 1.**
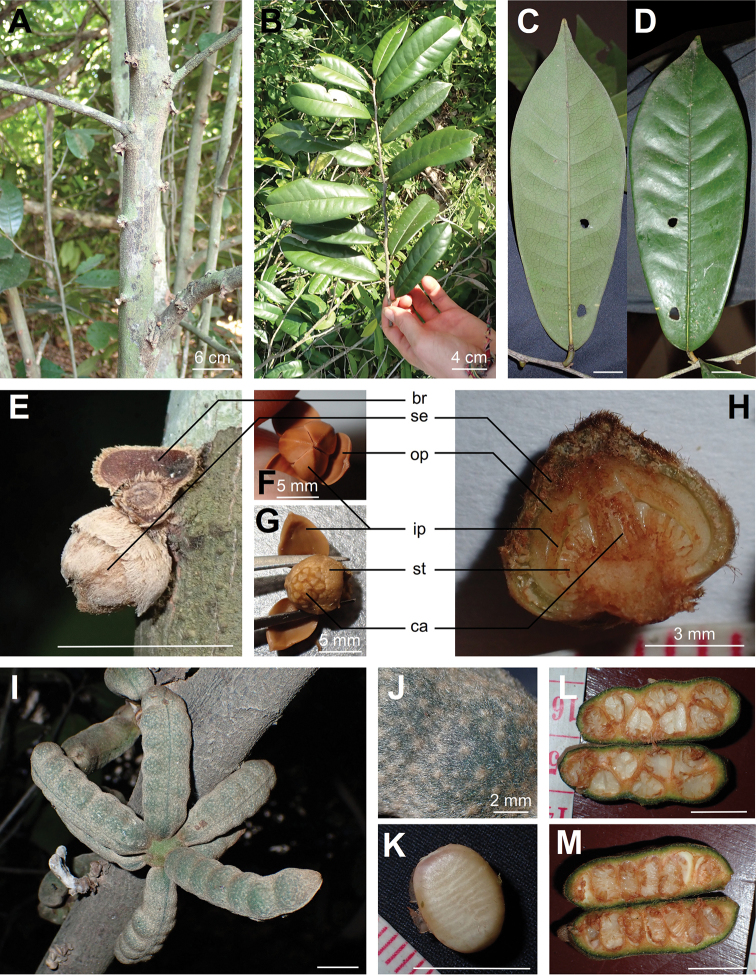
*Uvariodendron mbagoi***A** trunk with flower buds **B** young branch **C, D** entire leaf: **C** lower side **D** upper side **E–H** pre-anthetic flower bud: **E** on trunk (bottom) and fallen flower bud (top) **F** seen from top with sepals removed **G** seen from top with outer petals removed **H** longitudinal section; *br* bract, *ca* carpel, *ip* inner petal, *op* outer petal, *se* sepal, *st* stamen **I–M** Fruit: **I** entire with 7 monocarps **J** indumentum **K** seed **L** tangential cut **M** longitudinal cut. Photos by L.-P. M.J. Dagallier from the specimens U. Bloesch s.n. (**F, G**), L.-P.M.J. Dagallier 39 (**B, E, H, J, L**), 40 (**A, C, D**) and 50 (**I**). Scale bars: 10 mm unless stated.

#### Habitat.

Closed evergreen forest dominated by *Scorodophloeus fischeri*, on coral rag soil. Altitude: 90–340 meters.

#### Distribution.

**Figure 2.**
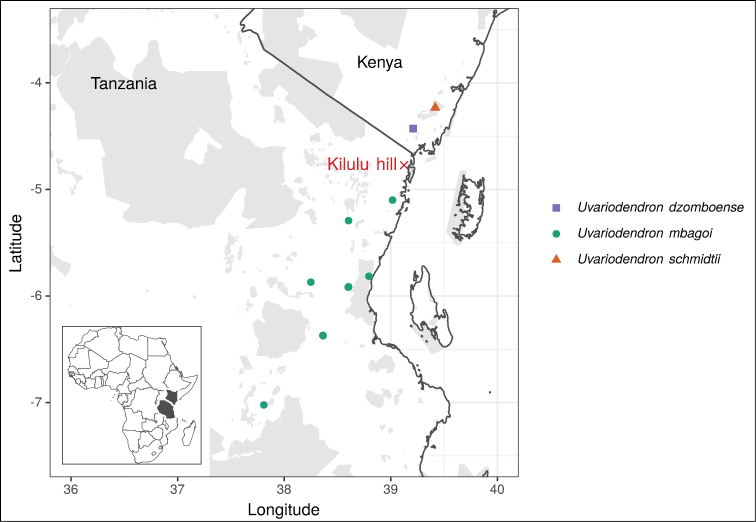
Map of the distribution of the three new *Uvariodendron* species. Protected areas are represented in grey shades (see Material and Methods for details). The red cross indicates Kilulu hill, where *Uvariodendron dzomboense* was expected to occur but was not found.

Endemic to Tanzania; only known from seven locations: Kimboza Forest, Msata Hill, Kwedijela forest, Kwedivikilo sacred forest, Mkwaja Ranch, Mkulumuzi river, and Hale (Fig. 2).

#### Conservation status.

This species is known from 11 records in seven locations. The current occurrence of the species in Mkulumuzi river and Hale is really unlikely given that these are now (sub)urban areas and that these records date back, respectively, more than 30 years and over a century. Changes in traditional practices and exploitation of traditionally protected forests had been observed more than 20 years ago (Mwihomeke et al. 1998). This indicates that the current occurrence of the species in Kwedivikilo sacred forest is uncertain. Kwedijela forest is a locality under deforestation pressure with local crops slowly gaining ground (L–P.M.J. Dagallier and F. Mbago, field observations). However, the occurrences of the species in Kwedijela forest are 500 meters from the limit of Saadani National Park (SNP), so the species is likely to occur within the SNP where the protection is strict. The only record of this species occurring within a protected area is in Kimboza Forest Reserve, which has been threatened by encroachment, logging and invasion by the exotic *Cedrela odorata* L. (Hall and Rodgers 1986, Patrick 2008).

For the reasons explained above, we removed the occurrences in Mkulumuzi river and Hale from the calculations of extent of occurrence (EOO) and the area of occupancy (AOO). Considering the five remaining localities, the EOO is 3867 km^2^ and AOO is 20 km^2^. Following IUCN criterion B (IUCN 2012), *Uvariodendron mbagoi* is therefore assigned a preliminary status of Endangered EN B1ab(i,ii,iii,iv)+2ab(i,ii,iii,iv).

#### Vernacular names.

Zigua (or Chizigua) language: Mchenene, Msenene (C.M. Kisena 3039), Mkenene (T.L.P. Couvreur 3, L–P.M.J. Dagallier 39, F. Mbago 3323).

#### Uses.

The bark is used as a spice for meat meals and for tea.

#### Additional field notes.

Foodplant of *Graphium kirbyi* (Papilionidae) (T.C.E. Congdon 532).

#### Etymology.

Named after Mr. Frank Mbago, curator of the Dar es–Salaam University herbarium (DSM), to whom we owe the discovery of this species, and in honor of his botanic knowledge and fieldwork expertise in Tanzania, in particular of Annonaceae. He is also co-discoverer of the endemic Tanzanian genus *Mwasumbia* (Couvreur et al. 2009).

#### Paratypes.

Tanzania – Morogoro • L.-P.M.J. Dagallier 50 (DSM, K, MO, MPU (MPU1379109), P, WAG); Morogoro Rural District, Kimboza forest; 7°01'18.38"S, 37°48'32.13"E; alt. 267 m; 15 Nov. 2019. – Pwani • U. Bloesch s.n. (WAG (WAG.1549674; WAG.1418750), Kwedijela Coastal Forest, T3; 5°55'00"S, 38°36'00"E; 18 Sep. 2004. • T.L.P. Couvreur 3 (DSM, WAG); Bagamoyo District, Mazizi hill, on road between Chilinze and Wami River; 6°22'14.4"S, 38°21'51"E; alt. 100 m; 09 Nov. 2006. • L.-P.M.J. Dagallier 1 (DSM, K, MO, MPU (MPU1379043, MPU1379066), P, WAG), Msata Hill, 30 km North of Chalinze; 6°22'17.78"S, 38°21'49.97"E; alt. 317 m; 06 Nov. 2019. – Tanga • T.C.E. Congdon 532 (K); Pangani District, Mkwaja Ranch; 5°48'50.76"S, 38°47'40.92"E; alt. 90 m; 04 Dec. 1998. • L.-P.M.J. Dagallier 40 (DSM, K, MO, MPU (MPU1379099), P, WAG); Handeni District, Kwedijela forest, ~8 km Kwamsisi village; 5°54'50.77"S, 38°36'13.27"E; alt. 155 m; 13 Nov. 2019. • W.D. Hawthorne 1420A (K); Tanga District, Mkulumuzi river, karst river valley, Steinbruch reserve; 5°06'00"S, 39°01'00.12"E; 12 Aug. 1982. • C.M. Kisena 3039 (MO); Handeni District, Collected from Kwedivikilo sacred forest near Manga Village; 5°06'00"S, 30°37'00"E; 17 Nov. 1997. • F.M. Mbago 3323 (DSM, K); Handeni District, Kwedijela forest, ~8 km Kwamsisi village; 5°54'50.77"S, 38°36'13.27"E; 07 Oct. 2004. • G.A. Peter 52283 (B, WAG, K), Inseln des Pangani bei Hale; 5°17'34.8"S, 38°36'14.06"E; alt. 340 m; 31 Jan. 1915.

#### Discussion.

*Uvariodendron mbagoi* is unique within *Uvariodendron* for the strong bergamot (*Citrus bergamia* Risso) scent of the crushed leaves and bark. This scent is between lemon and orange scent. Other African Annonaceae species present strong scents. For example, *Uvariodendron anisatum* Verdcourt (Verdcourt 1955) presents an aniseed scent, and *Uvariodendron molundense* (Diels) R.E.Fries *var. citrata* Le Thomas (Le Thomas 1969) and *Uvariopsis citrata* Couvreur & Niangadouma (Couvreur and Niangadouma 2016), present a lemon scent. However, no bergamot scent has been reported so far in Annonaceae.

The globose flower buds of this species easily fall off. Only flower buds were observed for this species, thus it is hard to infer the size of mature flowers. In the description above, the measures on the sepals and the petals are based on the dissection of the biggest flower bud of U. Bloesch s.n., and the carpel measurements were based on an old flower of T.C.E. Congdon 532 which has lost sepals and petals.

The fruiting specimens observed were collected from September to December. Collecting this species earlier in the year might permit the observation of flowers at anthesis.

### Uvariodendron dzomboense

Taxon classificationPlantaeMagnolialesAnnonaceae

Dagallier, W.R.Q. Luke & Couvreur sp. nov.

026836F4-FC5B-5EA0-B77B-992F85C00FD1

urn:lsid:ipni.org:names:77215718-1

Fig. 3

#### Type.

Kenya – Coast • S.A. Robertson et al. Mrima Dzombo Expedition 207 (holotype: K, isotypes: EA, MO, WAG), Kaya Dzombo Hill; 4°25'48"S, 39°12'36"E; alt. 300 m; 07 Feb. 1989.

#### Diagnosis.

This species differs from other *Uvariodendron* species by its 50–75 carpels that are densely pubescent and its leaves smaller than 150 mm in length and narrowly elliptic to elliptic. It differs from *U. kirkii* by its smaller leaves (132 mm maximum versus 210 mm maximum) and higher number of carpels (50–75 versus 7–20) (Table 1).

**Figure 3.**
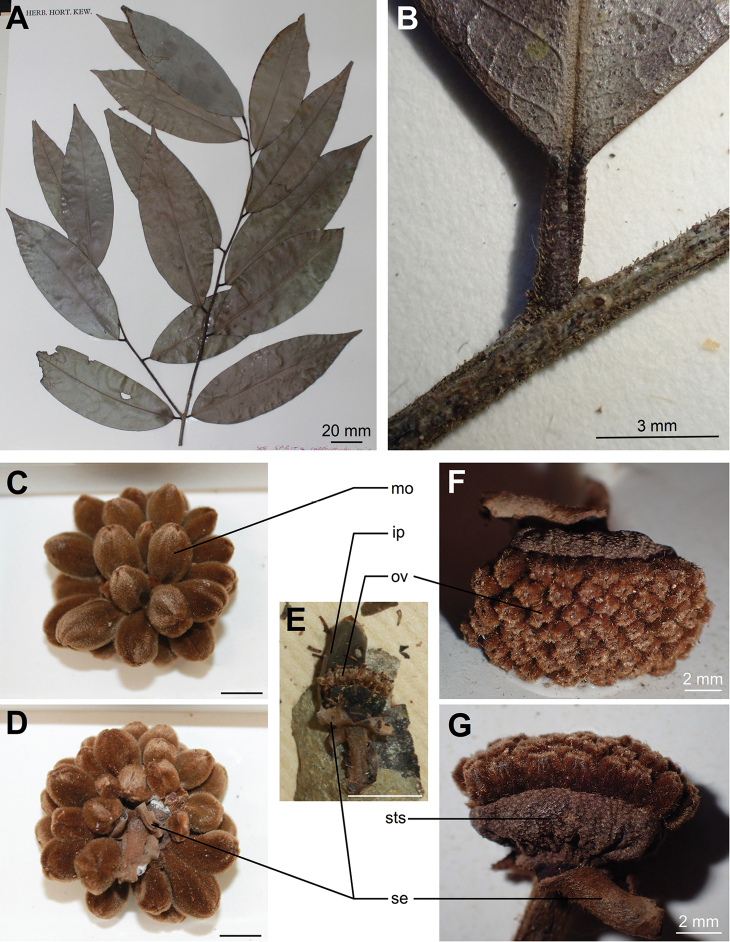
*Uvariodendron dzomboense***A** young branch with leaves **B** petiole and young branch detail **C, D** young fruit: **C** apical view **D** from the side **E–G** old flower: **E** entire on trunk **F** from top with details of ovaries **G** close-up. *ip* inner petal, *mo* monocarp, *ov* ovary, *se* sepal, *sts* stamen scars. Photos by L.-P. M.J. Dagallier from the specimens W.R.Q. Luke 1654 (**F, G**), W.R.Q. Luke 7443 (**B**) and S.A. Robertson MDE 207 (**A, C, D, F**). Scale bars: 10 mm unless stated.

#### Description.

Tree 4–7 m tall, d.b.h. unknown, young branches sparsely pubescent to glabrous, old branches glabrous. Leaf bud ‘eragrostiform’, composed of 5, distichous, longitudinally folded, velutinous scales. Leaves distichous, simple, entire, pinnately veined. Petiole 3–4 mm long, 1–1.5 mm in diameter, slightly pubescent to glabrous. Lamina 65–132 mm long, 20–45 mm wide, length:width ratio 2.9–3.6, narrowly elliptic to elliptic, coriaceous, apex attenuate, base acute to slightly decurrent, above glabrous, below glabrous when young and old; midrib sunken above, raised below, above glabrous when young and old, below slightly pubescent to glabrous when young, glabrous when old; secondary veins 12–13 pairs, weak brochidodromous; tertiary veins reticulate. Inflorescence borne on trunk or old branches, 1-flowered. Flowering pedicel 8–30 mm long, 2–2.5 mm in diameter, densely pubescent. Flowers actinomorphic, hermaphroditic, buds spherical 4–4.5 mm in diameter, sparsely pubescent. Bracts 6 at base of the pedicel in flower bud, 1 on mature flower in the lower half of the pedicel, 5–6 mm long, 5–8 mm wide, pubescent to shortly pubescent outside, glabrous inside. Sepals 3, 5–7 mm long, 4.5–7 mm wide, fused at base, pubescent to shortly pubescent outside, glabrous inside. Outer petals 3, ca. 16 mm long, ca. 9 mm wide, shortly velutinous outside, glabrous inside, color unknown. Inner petals 3, ca. 18 mm long, 8 mm wide, shortly velutinous outside, glabrous inside, color unknown. Stamens more than 700, 2 mm long, 0.5 mm wide, anthers linear, connective truncate. Carpels 50–75, ca. 2 mm long, ca. 1–1.5 mm wide, densely pubescent. Stigma not seen. Fruiting pedicel ca. 14 mm long, ca. 4 mm in diameter, pubescent. Monocarps (unripe?) ca. 35, ca. 15 mm long, ca. 10 mm wide, length:width ratio ca. 1.5, ovoid, sessile, densely pubescent, golden brown. Seeds (unripe?) ca. 5 per monocarp, uniseriate, ca. 4.5 mm long, ca. 1 mm wide, glabrous.

#### Distribution.

Endemic to Kenya, only known from Dzombo Hill (Kaya Dzombo) (Fig. 2).

#### Habitat.

Moist semi–deciduous forest on igneous intrusion.

#### Conservation status.

This species is known from five collections from a single location. Literature found on the Dzombo Hill forest reports a surface of 2.95 km^2^ (Critical Ecosystem Partnership Fund 2005). We calculated a surface of 5.31 km^2^ for the forested part of the hill (see Material and Methods for details). EOO and AOO are thus estimated at less than 5.40 km^2^. The Kaya Dzombo forest is gazetted as a sacred forest under the National Museums protection as a National Monument. However, the forest suffers from local logging for timber, poles and firewood, and has been impacted by fire on several occasions (W.R.Q. Luke, personal observations). Following IUCN criterion B (IUCN 2012), *Uvariodendron dzomboense* is therefore assigned a preliminary status of Endangered EN B1ab(iii)+2ab(iii).

#### Etymology.

The specific epithet comes from the Dzombo Hill where the species is endemic.

#### Paratypes.

Kenya – Coast • W.R.Q. Luke 1654 (EA (EA000008806), K); Kwale District, Dzombo Forest Reserve; 4°25'48"S, 39°12'36"E; alt. 270 m; 06 Jan. 1989. • W.R.Q. Luke et al. 2884 (EA, K); Kwale District, Dzombo Forest Reserve; 4°25'48"S, 39°12'36"E; alt. 270 m; 04 Oct. 1991. • W.R.Q. Luke et al. 3370 (EA); Kwale District, Dzombo Forest Reserve; 4°25'48"S, 39°12'36"E; alt. 270 m; 12 Nov. 1992. • W.R.Q. Luke & M. Pakia 7443 (K, EA (EA000008810)); Kwale District, Dzombo; 4°25'48"S, 39°12'36"E; alt. 270 m; 28 Jun. 2001.

#### Discussion.

This species is known as “*Uvariodendron sp. nov. 1 of CFS*” in the annotated checklist of the coastal forests of Kenya (Ngumbau et al. 2020).

The only fruit known from this species (Robertson S.A. et al. MDE 207) presents ca. 35 ovoid monocarps. These are densely pubescent and have small seeds compared to other *Uvariodendron* species (4.5 mm long vs. 8–20 mm long). However, it is unclear whether this observed fruit is ripe or not. Further collections could bring more information. This species also presents an ‘eragrostiform’ leaf–bud (see discussion of *Uvariodendron schmidtii* below).

During a field trip in Tanzania in November 2019, we explored the forest of Kilulu hill (TANZANIA- Tanga, 4°46'22"S, 39°07'30"E, see Fig. 2). Kilulu hill is ca. 40 kilometers as the crow flies south from Dzombo hill were *U. dzomboense* occurs. We expected to find *U. dzomboense* there but our quest on every slope of the hill was unsuccessful. This indicates that the dispersal distance of *U. dzomboense* might be very short.

### Uvariodendron schmidtii

Taxon classificationPlantaeMagnolialesAnnonaceae

W.R.Q. Luke, Dagallier & Couvreur sp. nov.

9DBB2C68-311D-5E19-833C-135915980B9E

urn:lsid:ipni.org:names:77215719-1

Figure 4

#### Type.

Kenya – Coast • W.R.Q. Luke 3087 (holotype: EA (EA000008814), isotypes: K, MO, US); Kwale District, Shimba hills, Longomagandi; 4°14'00"S, 39°25'00"E; alt. 380 m; 20 Apr. 1992.

#### Diagnosis.

This species differs from other *Uvariodendron* species by its flowers that are small (petals < 13 mm long), velutinous, on a 10–15 mm long pedicel, with fused sepals forming a ring around the fruit pedicel, and fewer than 10 carpels. It differs from *U. kirkii* by its smaller petals (< 13 mm versus more than 15 mm) and its sepals fused in a ring (versus free and valvate to imbricate) (Table 1).

**Figure 4.**
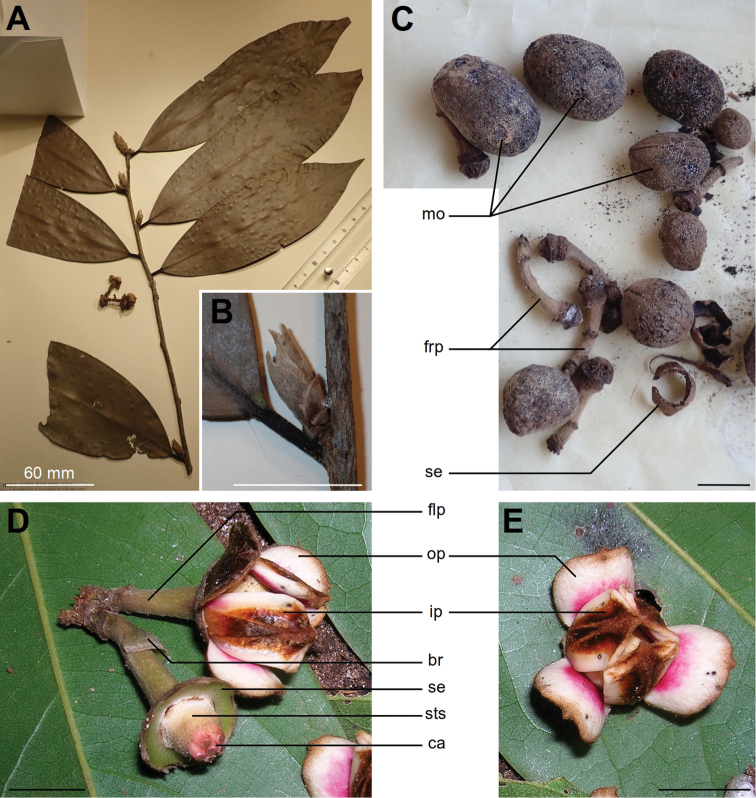
*Uvariodendron schmidtii***A** young branch with leaves **B** eragrostiform axillary bud **C** detached monocarps and pedicels **D** two-flowered inflorescence **E** flower, apical view. *br* bract, *ca* carpel, *flp* flower pedicel, *frp* fruit pedicel, *ip* inner petal, *mo* monocarp, *op* outer petal, *se* sepals ring, *sts* stamen scars. Photos by L.-P. M.J. Dagallier from the specimens W.R.Q. Luke 3087 (**C**) and W.R.Q Luke 4717 (**A, B**), and by W.R.Q. Luke from a living individual (**D, E**). Scale bars: 10 mm unless stated.

#### Description.

Tree 10–12 m tall, d.b.h. unknown, young branches sparsely pubescent to glabrate, old branches glabrous. Leaf bud ‘eragrostiform’, composed of 5–7, ca. 10 mm long, 10 mm wide distichous, longitudinally folded, velutinous scales. Leaves distichous, simple, entire. Petiole 4.5–7 mm long, 1.5–2 mm in diameter, glabrate to sparsely puberulent. Lamina 159–188 mm long, 49–71 mm wide, length:width ratio 2.4–3.3, narrowly elliptic to elliptic, coriaceous, apex attenuate to acuminate, base acute to decurrent, above glabrous, below sparsely pubescent to glabrate when young, glabrous when old; midrib sunken above, raised below, above glabrous when young and old, below pubescent to glabrous when young, glabrous when old; secondary veins 10–14 pairs, weakly brochidodromous to brochidodromous; tertiary veins reticulate. Inflorescence borne on trunk and branches, 1–2 flowers. Flower pedicel 10–15 mm long, 2.5 mm in diameter, densely velutinous. Flowers actinomorphic, hermaphroditic, buds spherical, 6–7 mm in diameter, velutinous. Bracts 1–3, 1 at base of the pedicel, 1–2 between the 20–70% of the length of the pedicel, ca. 5 mm long, ca. 10 mm wide, velutinous outside, glabrous inside. Sepals 3, 5.5–7 mm long, 7–9 mm wide, fused on ca. 50% of the length, forming a ring around fruit pedicel, densely velutinous to velutinous outside, glabrous inside. Outer petals 3, 11–12 mm long, 9–11 mm wide, densely velutinous to velutinous outside, glabrous inside, brown outside, cream with purple streak at base inside. Inner petals 3, ca. 10 mm long, 8–9 mm wide, connivent at apex on ca. 50% of the length, densely velutinous to velutinous outside, glabrous inside, brown-orange with margins cream and purple at base outside, cream with purple streak at base inside. Stamens more than 500, length and shape unknown. Carpels ca. 7, ca. 1.5 mm long, ca. 1 mm wide, velutinous. Stigma not seen. Fruiting pedicel ca. 16 mm long, ca. 2.5 mm in diameter, pubescent. Monocarps 3–5, ca. 32 mm long, ca. 20 mm wide, length:width ratio ca. 1.6, rounded to ellipsoid with a longitudinal ridge, sessile, sparsely pubescent, green turning orange. Seeds not seen.

#### Distribution.

Endemic to Kenya; only known from the Longomwagandi forest (also found spelled “Longomagandi” or “Longo-Magandi” in the literature) in the Shimba Hills National Reserve, in Kenya (Fig. 2).

#### Habitat.

Lowland forest on ridge with *Antiaris*, *Milicia*, *Lovoa*, *Celtis*, *Quassia*, *Hymenaea*, *Julbernardia*, *Diospyros*, *Memecylon*, and many Rubiaceae shrubs in understorey.

#### Conservation status.

This species is known from seven collections from a single location. Literature for the Shimba Hills forest reserve reports a surface between 0.22 km^2^ (Schmidt 1992) and 1.50 km^2^ (Cheek 2003) for Longomwagandi forest. We calculated a surface of 1.30 km^2^ (see Material and methods for details). EOO and AOO are thus estimated at less than 1.50 km^2^. Following IUCN criterion B, this would place the species in the “Critically Endangered CR” category. However, given that the occurrences are in the Shimba Hills National Reserve, no decline is observed or projected in EOO and AOO. The future of *Uvariodendron schmidtii* relies on the future of Shimba Hills National Reserve. Given that the species occurs in a single locality with a very restricted AOO (less than 20 km^2^), it is “prone to the effects of human activities or stochastic events within a very short time period in an uncertain future, and is thus capable of becoming Critically Endangered or even Extinct in a very short time period” (IUCN 2012). Following IUCN criterion D, it can be therefore assigned a preliminary status of Vulnerable VU.

Copious seedlings are found below parent trees, but few survive to maturity. Many of these “wildlings” were moved to the Base Titanium indigenous tree nursery and have been out-planted there as part of their mine rehabilitation program.

#### Vernacular name.

Mbebeneka in Kidigo language (R. Schmidt 788).

#### Etymology.

This species is named after Robert Schmidt, a PhD student studying the ecology of the Shimba Hills National Reserve who first collected it in September 1988 and brought it to the attention of W.R.Q. Luke.

#### Paratypes.

Kenya – Coast • W.R.Q. Luke & S.A. Robertson 2737 (EA, K, MO, US); Kwale District, Shimba hills, Longomagandi; 4°14'00"S, 39°25'00"E; alt. 390 m; 18 Mar. 1991. • W.R.Q. Luke 2919 (EA (EA000008817), K, MO, US); Kwale District, Shimba hills, Longomagandi; 4°14'00"S, 39°25'00"E; alt. 390 m; 15 Oct. 1991. • W.R.Q. Luke 4717 (P (P02084012), Ukunda); Kwale District, Shimba hills, Longomwagandi; 4°14'00"S, 39°25'00"E; alt. 380 m; 12 Sep. 1997. • W.R.Q. Luke 11676 (EA, K, MO, US); Kwale District, Shimba hills, Longomagandi; 4°14'00"S, 39°25'00"E; alt. 380 m; 30 Dec. 2006. • S.A. Robertson 7556 (EA, K, WAG (WAG0129164)); Kwale District, Shimba Hills, Longomagandi; 4°14'00"S, 39°25'00"E; alt. 450 m; 04 Jun. 2005. • R. Schmidt 788 (EA); Kwale District, Shimba Hills, Longomagandi; 07 Sep. 1988.

#### Discussion.

This species shows ‘eragrostiform’ leaf–buds, a feature described in *Uvariodendron gorgonis* Verdcourt (Verdcourt 1969) and *Uvariodendron dzomboense* Dagallier, W.R.Q. Luke & Couvreur (this publication). This structure is composed of several (5–7 in *U. schmidtii* and *U. dzomboense*, 6–12 in *U. gorgonis*) distichous and densely pubescent scales that might be a protection for the apical meristem against drought or herbivores. The adjective ‘eragrostiform’ refers to the genus *Eragrostis* (Poaceae) that has a peculiar form of flattened spikelet composed of compact and clustered florets. Even though this feature is striking, it seems hard to use it as a diagnostic character. Similar apical buds are also found in other Annonaceae species such as *Monodora minor* Engler & Diels (Couvreur 2009) or in *Uvariodendron usambarense* Fries and *Uvariodendron giganteum* (Engler) Fries.

### Polyceratocarpus oligocarpus

Taxon classificationPlantaeMagnolialesAnnonaceae

(Verdc.) Dagallier comb. nov.

D0B89BE8-3FB4-5B15-9E53-3C082F51EC73

urn:lsid:ipni.org:names:77215720-1

Uvariodendron oligocarpum Verdcourt, Kew Bulletin 41(2): 289, 1986.

#### Type.

Tanzania – Tanga • J. Lovett 259 (holotype: K (K000198888)); Lushoto District, Ambangulu, West Usambara; alt. 1300 m; 2 Mar. 1984.

We examined 11 specimens (including the type specimen) of *Uvariodendron oligocarpum* Verdc. and found they have percurrent tertiary venation and pitted seeds. These characteristics are typical of the genus *Polyceratocarpus* Engl. & Diels (Couvreur et al. 2009, 2012). Moreover, the fertile specimens observed have the combination of the following characters: outer petals ca. 35 mm long, 3 to 6 carpels, and 2 to 4 cylindrical and straight to slightly curved monocarps. This combination precludes these specimens from being identified as one of the two other species known from East Africa to date: *Polyceratocarpus scheffleri* Engl. & Diels that has “at least 20 [and] strongly curved” monocarps (Verdcourt 1971), and *Polyceratocarpus askhambryan-iringae* A.R. Marshall & D.M. Johnson that has outer petals 10–16 mm long (Marshall et al. 2016). Based on the above characters, they also cannot be included in any other accepted species from Central or West Africa that all have petals shorter than 25 mm long: *Polyceratocarpus angustifolius* Paiva and *P. germanii* Boutique, *P. gossweileri* (Excell) Paiva, *P. laurifolius* Paiva, *P. microtrichus* (Engl. & Diels) Ghesq. ex Pellegr., *P. parviflorus* Ghesq., and *P. pellegrinii* Le Thomas (Pellegrin 1949, Boutique 1951, Le Thomas 1965, Paiva 1966). Thus, this species initially described as *Uvariodendron oligocarpum* Verdc. is here combined as *Polyceratocarpus oligocarpus* (Verdc.) Dagallier.

#### Other specimen examined.

Tanzania – Tanga • A. Borhidi 86249 (K); Muheza District, East Usambaras Mts., Kwamkoro F.R. SE of Kwamkoro Tea Estate; alt. 1030 m; 28 Oct. 1986. • A. Borhidi 87241 (K); Muheza District, East Usambaras Mts., Kwamkoro F.R. bordering Kwamsambia F.R; alt. 990 m; 05 May. 1987. • L.-P.M.J. Dagallier 63 (DSM, K, MPU (MPU1379122), P, WAG); Korogwe District, East Usambaras, Ambangulu, top of the mountain above the tea plantations; 5°04'13.00"S, 38°24'31.00"E; alt. 1320 m; 20 Nov. 2019. • A.R. Marshall 1457 (K, MO); Lushoto District, Ambangulu – PSP19, Ambangulu Tea Estate Forest, West Usambara Mountains; 5°4'20.69"S, 38°24'24.21"E; alt. 1294 m; 22 Mar. 2008. • A.R. Marshall 1695 (K); Lushoto District, Ambangulu – PSP19, Ambangulu Tea Estate Forest, West Usambara Mountains; 5°4'20.69"S, 38°24'24.21"E; alt. 1294 m; 24 Mar. 2008. • F.M. Mbago 3586 (DSM); Lushoto District, Balangai forest near Tea estate; 4°56'41.24"S, 38°26'42.10"E; alt. 1505 m; 28 Jul. 2012. • F.M. Mbago 3760 (DSM), Korogwe Kunga Forest Mavimo Kwemtonto; 3°20'37.15"S, 37°19'46.06"E; alt. 898 m; 23 Nov. 2016. • C.K. Ruffo 1730 (K); Muheza District, Kwamkoro F.R; alt. 950 m; 28 Oct. 1986. • C.K. Ruffo 1747 (K); Muheza District, Kwamkoro F.R; alt. 1000 m; 31 Jan. 1987. • C.K. Ruffo 1835 (K); Muheza District, Kwamkoro F.R; alt. 1050 m; 18 Sep. 1986.

### Key to the East African species of *Uvariodendron*

**Table:** 

1	Longest leaf lamina equal to or longer than 35 cm long	**2**
–	Longest leaf lamina shorter than 35 cm long	**4**
2	Number of secondary veins pairs equal to or less than 20; fruit monocarps less than 10 mm wide and with a length:width ratio over 5, stipe 5–11 mm long	***U. gorgonis* (pro parte)**
–	Number of secondary veins pairs more than 20; fruit monocarps more than 13 mm wide and with a length:width ratio below 4, stipe less than 6 mm long	**3**
3	Young branches sparsely pubescent to glabrous; leaf lamina oblong to obovate, base rounded to subcordate	***U. usambarense***
–	Young branches pilose covered with long soft hair quickly falling off; leaf lamina obovate, base acute	***U. magnificum***
4	Greatest leaf lamina equal to or shorter than 16 cm long, margins slightly revolute	**5**
–	Greatest leaf lamina longer than 16 cm long, margins flat	**6**
5	Bark and crushed leaves emitting a strong bergamot scent; flower and fruits (sub)sessile, pedicel less than 6 mm long; carpels 12 to 16; monocarps cylindrical, green-grey, tomentose with regular tufts of higher hair density	***U. mbagoi***
–	Bark and crushed leaves not emitting a bergamot scent; flower and fruits pedicel 8–30 mm long (but flower buds sessile); carpels 50 to 75; monocarps ovoid, golden-brown, densely pubescent	***U. dzomboense***
6	Leaf lamina up to 40 cm long, base rounded to acute; carpels 40 to 50; monocarp length:width ratio over 5	***U. gorgonis* (pro parte)**
–	Leaf lamina up to 32 cm long, base acute to decurrent; carpels up to 40; monocarp length:width ratio below 4	**7**
7	Leaf lamina apex attenuate to acuminate; flower pedicel equal to or less than 15 mm; sepals fused at base over more than 20% of their length	**8**
–	Leaf lamina apex acute to attenuate; flower pedicel equal to or more than (5)-10 mm; sepals connivent or fused at base over less than 10% of their length	**9**
8	Bark of trunk and branch peeling off, reddish; leaf lamina length:width ratio equal to or more than 3.4; petals 31–36 mm long, carpels 29 to 40	***U. pycnophyllum***
–	Bark of trunk and branch not peeling off, greyish; leaf lamina length:width ratio equal to or less than 3.3; petals 10–12 mm long, carpels fewer than 10	***U. schmidtii***
9	Plant emitting a strong anise scent; longest leaves up to 32 cm; flower pedicel 15–65 mm long; fruit monocarps 38–70 mm long, ripe fresh fruit dark blue-black	***U. anisatum***
–	Plant not emitting anise scent; longest leaves up to 22 cm; flower pedicel (5)10–30 mm long; fruit monocarps 23–36 mm long, ripe fresh fruit dull-orange	***U. kirkii***

## Discussion

The three new species described here (*Uvariodendron mbagoi* Dagallier & Couvreur, *Uvariodendron dzomboense* Dagallier, W.R.Q. Luke & Couvreur and *Uvariodendron schmidtii* W.R.Q. Luke, Dagallier & Couvreur) occur in the coastal forests of Kenya and Tanzania. Due to their restricted ranges, they are all threatened following our IUCN preliminary conservation status assessments. Endemism is high in East Africa for plants and animals (Burgess et al. 1998, 2007), and single-location endemic species are known there (e.g. Scharff 1992, Couvreur et al. 2009, Gosline et al. 2019). The discovery of these three new narrowly endemic species supports this long-standing observation. This also shows that botanically this region is still not fully known despite the publication of the complete flora of East Africa (Beentje 2015). There are still new plant species that have been collected and await description (see e.g. “sp. nov.” for several genera in Ngumbau et al. 2020).

The new combination and the three new species described here bring the number of East African *Uvariodendron* species to nine across East Africa, and to 17 across Africa. The described species show characters that have never been found in the family, such as the strong bergamot scent of *Uvariodendron mbagoi* or in the genus, such as the very densely pubescent carpels and monocarps of *U. dzomboense*.

## Supplementary Material

XML Treatment for Uvariodendron mbagoi

XML Treatment for Uvariodendron dzomboense

XML Treatment for Uvariodendron schmidtii

XML Treatment for Polyceratocarpus oligocarpus
